# An Unusual Case of Denervation Changes of the Intercostal Muscles Associated with Intercostal Neuralgia in a Patient with Chest Pain

**DOI:** 10.15388/Amed.2024.31.1.4

**Published:** 2024-02-27

**Authors:** Rajesh Botchu, Lorraine Aspland, Sisisth Ariyaratne, James Burgess, Gurjit Bhogal, David Beale

**Affiliations:** 1 Department of Musculoskeletal Radiology, Royal Orthopaedic Hospital, Birmingham, UK; 2 Centre of Musculoskeletal Medicine, Royal Orthopaedic Hospital, Birmingham, UK; 3 Department of Radiology, Heath Lodge Clinic, Knowle, UK

**Keywords:** Denervation, post viral infection, intercostal neuralgia, rib pain, thoracic, MRI, Denervacija, ankstesnė virusinė infekcija, tarpšonkaulinė neuralgija, šonkaulių skausmas, krūtinės ląsta, MRI

## Abstract

Musculoskeletal aetiologies account for most patients presenting with chest pain. Intercostal neuralgia is a lesser-known cause of musculoskeletal chest pain, which can present a diagnostic challenge with nonspecific imaging findings. We report a case of a 31-year-old male who presented with severe lower thoracic and chest wall pain following a suspected viral infection, where Magnetic Resonance Imaging (MRI) revealed characteristic features of denervation oedema within the affected intercostal muscles. This pattern of imaging findings in intercostal neuralgia is sparely described in the current literature. MRI along with history and examination was crucial in diagnosing the condition and excluding other potential causes of musculoskeletal chest wall pain on this occasion. The patient’s symptoms were subsequently managed conservatively. The case highlights the importance of considering intercostal neuralgia as a potential cause of chest wall pain, particularly in the setting of post viral infection and absence of preceding mechanical musculoskeletal injury and explores an uncommon yet characteristic imaging finding which may be important in diagnosing the condition.

## Abstract (it)

Nustatyti raumenų ir skeleto skausmų priežastį yra aktualu daugumai pacientų, besikreipiančių dėl skausmo krūtinės plote. Tarpšonkaulinė neuralgija yra nedažna raumenų ir skeleto krūtinės srities skausmų priežastis. Ją nustatyti iš gaunamos vaizdinės informacijos gali būti sunku, nes joje nėra jokių išskirtinių požymių.

**Atvejo pristatymas:**

31 metų vyras kreipėsi dėl intensyvaus skausmo apatinėje krūtinės ląstos dalyje bei krūtinės sienoje. Skausmas atsirado po, kaip spėjama, virusinės infekcijos. Magnetinio rezonanso tyrimas (MRI) atskleidė įprastinius denervacijos edemos požymius pažeistuose tarpšonkauliniuose raumenyse. Tokios tendencijos vaizdinėje medžiagoje tarpšonkaulinės neuralgijos atveju net ir naujausioje literatūroje yra menkai aprašytos. MRI, ligos istorija ir paciento apžiūra buvo itin svarbios nustatant skausmo priežastį ir atmetant kitas galimas raumenų ir skeleto krūtinės sienos skausmą sukeliančias priežastis šiuo konkrečiu atveju. Anksčiau paciento simptomai buvo išgydyti konservatyviais metodais. Šis atvejis atskleidžia, kaip svarbu atsižvelgti į tarpšonkaulinės neuralgijos galimybę kaip į galimą krūtinės sienos skausmo priežastį, ypač jei buvo persirgta virusine liga ir nėra žinoma buvus jokių ankstesnių mechaninių raumenų ar skeleto traumų. Šiuo atveju ištyrėme nedažną, tačiau vis dėlto standartinę diagnozę, gautą iš vaizdinės medžiagos, kuri gali būti svarbi nustatant šią sveikatos problemą.

## Introduction

Chest pain is a common presentation in the healthcare setting and carries a complex and broad differential diagnosis due to the numerous pathologies involved. [1] According to a recent study, musculoskeletal pain was the commonest cause of acute chest pain, accounting for 51.2% of the presentations. It may affect any of the chest wall structures including the ribs, intercostal muscles and the overlying skin and subcutaneous tissue. [2,3] Winzenberg et al. classified common musculoskeletal causes of chest pain into 3 groups: isolated chest wall musculoskeletal pathology including traumatic, mechanical, and inflammatory causes, such as fractures, slipping rib syndrome and costochondritis, rheumatological conditions including those affecting the thoracic spine, facet, and costovertebral joints, and nonrheumatological systemic causes including neoplasia. [1]

Intercostal neuralgia is an important yet underdiagnosed and undertreated cause of the chest wall pain. Diagnosis of the condition can be challenging due to overlap of symptoms with other more common causes of chest wall pain, inadequate imaging modalities and decreased awareness of the condition. [4] It has been established that viral infections have neurotropic and neuroinvasive capabilities which can affect both the peripheral and central nervous system, and as such viral infections are commonly responsible for the condition. [5]

We report a case of atypical unilateral chest wall pain in a young adult male after a suspected viral infection, with characteristic features of denervation oedema seen within the affected intercostal muscles on MRI. The patient was conservatively managed with regards to his symptoms.

This paper aims to make the reader aware of this uncommon yet important diagnosis of intercostal neuralgia with denervation changes within intercostal muscles as a potential cause of acute chest wall pain. While intercostal neuralgia is a known cause of chest wall pain, diagnostic imaging features of this condition are sparsely described in the existing literature. A study by Chalian et al. described the role of magnetic resonance neurography (MRN) in diagnosing this entity. [4] However denervation changes within the intercostal muscles as a feature has not been described in the existing literature, and a search conducted on the PubMed and Ovid Medline databases did not reveal any similar cases. As such, this would be a first reported case of this entity.

## Case Report

A 31-year-old male presented with a 13-week history of insidious, initially left-sided thoracic para-spinal pain which progressively extended to involve the left chest wall and lower ribs. He described hypersensitivity of this region to touch and pressure. Prior to the onset of these symptoms, he described a history of a generalised flu-like illness with fever. The exact aetiology was unknown but presumed to be a viral infection. He has a history of type-1 diabetes which was well-controlled with Insulin and continuous glucose monitoring. There was no history of trauma or other relevant medical or surgical history. He underwent physiotherapy, which exacerbated the symptoms.

On examination, there was no rash, blisters, or skin changes over the affected area. On palpation, there was marked widespread hypersensitivity and hyperalgesia of the left chest wall, but no bony tenderness. Normal mobility of the spine was noted without structural or postural deviation. Side lying quadratus lumborum stretch was normal bilaterally. Sit ups caused some side and abdominal pain.

Chest radiograph and thoracic spine MRI performed (T1 and STIR axial and coronal) following review with a spinal specialist revealed no abnormality. Specifically, there was no evidence of thoracic disc herniation or foraminal narrowing on MRI. A rheumatology opinion was then sought. Subsequent blood tests (full blood count, ESR, CRP and renal function) and Computed Tomography (CT) of the abdomen (looking for potential causes of referred pain) were also normal.

**Figures 1a and 1b:**
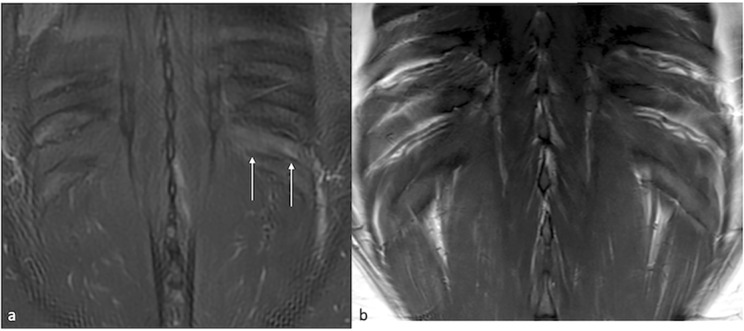
Coronal STIR image of chest wall (1a) demonstrating uniform high signal within the intercostal muscle layers between the left 10th and 11th ribs in keeping with oedema *(white arrows)*. Normal appearance of the adjacent ribs with no marrow signal change. Coronal T1 nonfat saturated image of chest wall (1b) demonstrating no evidence of fatty atrophy of the affected intercostal muscles.

**Figure 2:**
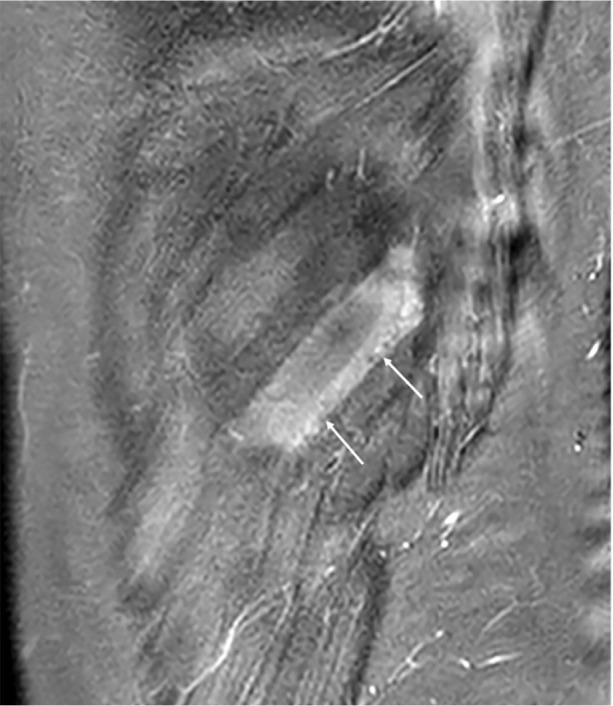
Sagittal STIR image of left chest wall demonstrating uniform high signal within the intercostal muscle layers between the left 10th and 11th ribs in keeping with oedema *(white arrows)*.

**Figure 3:**
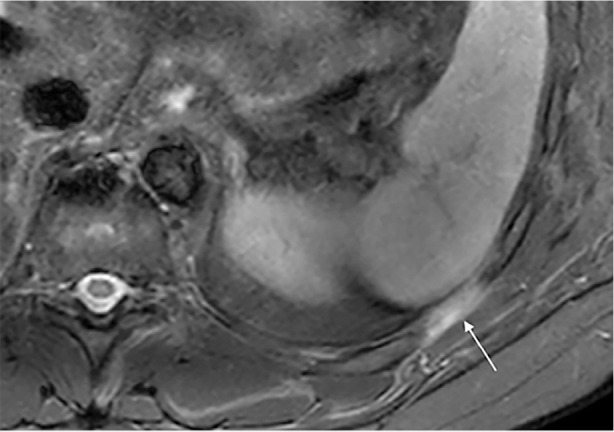
Axial STIR image of left chest wall demonstrating uniform high signal within the intercostal muscle layers between the left 10th and 11th ribs in keeping with oedema *(white arrows)*. Note homogenous involvement of all 3 intercostal muscle layers.

MRI of the chest wall was then arranged with a working diagnosis of post-infectious neuralgia and to rule out any other chest wall pathology. Fluid sensitive (T2 fat suppressed and STIR (short tau inversion recovery)) sequences on MRI demonstrated uniformly increased high signal within the internal, external, and innermost intercostal muscles between the left 10th and 11th ribs. There was no fatty atrophy of the muscles on the T1 weighted sequences. The adjacent 10th and 11th ribs were normal, with no evidence of fracture or marrow signal change *(Figures 1a, 1b, 2 and 3)*.

The diffuse involvement of the intercostal muscles was atypical for traumatic pathology, and a diagnosis of denervation change was made.

Viral titres including Epstein Barr Virus (EBV), Cytomegalovirus (CMV), SARS-CoV-2, Varicella Zoster (VZV), Influenza and Human Immunodeficiency Virus (HIV) performed subsequently were inconclusive. The patient’s symptoms were managed successfully conservatively with Amitriptyline and Naproxen.

## Discussion

Thoracic spine and chest wall pain can be a challenging condition to diagnose, and often extensive investigations are carried out. [1] A detailed history including the onset, location and nature of the pain and physical examination is essential in eliciting the cause of musculoskeletal chest wall pain once significant and potentially life-threatening visceral pathology has been excluded. Intercostal neuralgia is a less commonly seen cause of musculoskeletal chest wall pain.

Intercostal nerves are part of the somatic nervous system, and supply motor and sensory innervation to the chest wall and pleura. They arise from the anterior rami of the T1–T11 thoracic spinal nerves. They enter the corresponding intercostal space between the parietal pleura and the intercostal membrane, and course along the subcostal groove of the rib above. The major branches of a typical intercostal nerve include the rami communicantes, the muscular branches, the collateral branch, the lateral cutaneous branch, and the anterior cutaneous branch. The rami communicantes carry visceral signals. The muscular branches provide motor supply to the intercostal muscles as well as several other chest wall muscles. The collateral branches innervate the intercostal muscles, parietal pleura, and periosteum of ribs. Cutaneous branches supply sensory innervation to the skin. [6]

There are numerous causes of intercostal neuralgia. These include anatomical compression, trauma and following viral infection such as with varicella zoster virus (VZV). [7] The absence of an associated rash should not rule out reactivation of VZV. [8] Viral infections are known to have neurotropic and neuroinvasive actions. [5] Koyuncu et al. identified that these infections can spread into the peripheral nervous system. [9] Immune and inflammatory responses associated with viral infections can damage peripheral nerve fibres, which in turn can result in a lower threshold for action potentials, spontaneous discharge of the neurons, and disproportionate responses to stimuli. These can cause peripheral sensitization and pain in the absence of painful stimuli giving rise to the clinical manifestations of neuralgia. [10] Usually, the condition is self-resolving with time, postural adaptations, and physiotherapy. In cases where this is not the case, further exploration may be required.

Given our patient was young, healthy and did not have a history of a rash, a post herpetic neuralgia related to VZV reactivation was thought less likely. Other forms of neuralgia such as Parsonage–Turner syndrome, a form of brachial neuritis which has a similar pathophysiology and can cause denervation changes in the suprascapular nerve distribution, has been associated with preceding viral respiratory and influenza like illnesses as well as COVID-19 infection. [11, 12] Hence it is possible that some other form of viral infection could have resulted in this patient’s neuralgia, particularly given the history of previous flu-like illness.

The clinical presentation with hyperalgesia, radicular pattern of symptoms and history are useful diagnostic indicators which help distinguish this entity from other commonly seen musculoskeletal causes of chest wall pain. [1, 13] MRI plays a crucial role in diagnosing denervation changes related to neuralgia. Hyperintense signal changes within the affected muscle groups can be seen on fluid sensitive sequences. The observed denervation changes can also vary depending on the stage of the disease, and changes of fatty infiltration may be seen in subacute and chronic cases, best assessed on nonfat saturated T1 and T2 sequences.11 The diffuse and extensive involvement of all three intercostal muscle layers at the affected level in this patient would be atypical for traumatic injury such as a muscle strain. MRI was also useful in this setting in excluding more common causes of referred neuropathic pain in the chest wall due to thoracic disc herniation or vertebral foraminal narrowing, which can result in thoracic nerve entrapment and giving rise to a similar presentation. [1, 14]

## Conclusion

Intercostal neuralgia due to post viral denervation needs to be considered in the differential diagnosis of unilateral chest pain particularly with history of viral infection, in the absence of trauma and ruling out referral of visceral cause. The absence of a rash should not exclude this from consideration. MRI is a useful diagnostic tool in persisting or severe cases with a detailed history. Treatment other than pain medication and time is not usually indicated.

## Statements and declarations

The authors did not receive any funding for this work.
